# Use of gastric residual volume measured by nasogastric feeding tubes for management of dogs and cats after gastrointestinal foreign body surgery: a retrospective study

**DOI:** 10.3389/fvets.2026.1791629

**Published:** 2026-06-04

**Authors:** Amy Sheil, Elizabeth A. Stewart, George E. Moore, Marie E. Kerl, Melissa Tye

**Affiliations:** 1 VCA Animal Referral and Emergency Center of Arizona, Mesa, AZ, United States; 2 Mars Veterinary Health Science, Vancouver, WA, United States

**Keywords:** enterectomy, enterotomy, gastric residual volume, gastrotomy, ileus, nasogastric tube

## Abstract

Gastrointestinal foreign body obstruction is a frequently diagnosed ailment in small animal medicine and postoperative ileus can be a challenging sequela to manage. Nasogastric feeding tubes can be utilized after surgery for both quantification of gastric residual volumes to identify ileus as well as nutritional management in anorexic patients. This single-institution retrospective study assesses the association of gastric residual volumes measured via nasogastric feeding tubes with gastrointestinal signs and complications in dogs and cats undergoing foreign body surgery, with and without temporary nasogastric tube placement for postoperative management. In this retrospective study, 469 dogs and cats that had foreign body surgery within a 3-year period were included. These were arranged into two groups for analysis, 210 patients that had a nasogastric tubes placed and 259 that did not. Signalment, presenting clinical signs, and surgical procedure(s) performed were similar between groups, however, nasogastric tube placement was more likely for patients that underwent enterectomy (85.2%, *p* < 0.001). Total average gastric residual volume in the first 12 h after surgery was 2.0 mL/kg (range 0.0–38.9 mL/kg) and was 5.4 mL/kg (range 0.0–64.1 mL/kg) for the first 24 h. Frequency of upper gastrointestinal signs associated with postoperative ileus (vomiting and regurgitation) did not statistically differ between groups (14.4% vs. 14.7%, *p* = 0.118), however, with statistical analysis controlled for drug administration, the odds of vomiting and regurgitation were reduced approximately 50–60% for patients having nasogastric tubes placed and aspirated consistently (every 4–6 h) compared to those without. A gastric residual volume cut of >12 mL/kg over the first 24-h period after gastrointestinal foreign body surgery was most significantly associated with increased risk of vomiting and regurgitation (*p* = 0.058). Nasogastric tube placement was associated with substantially longer hospital stays after surgery (*p* < 0.001). Cats were less likely to have a feeding tube placed compared to dogs (35.5% vs. 47.1%) and were significantly less likely to regurgitate than dogs (1.1% vs. 17.6%, *p* = 0.001) despite some having higher gastric residual volumes. Additionally, usage of methadone in the perioperative period was associated with lower frequency of vomiting and regurgitation (*p* = 0.024). There was a low rate of complications associated with nasogastric tube placement noted in this study (2.9%).

## 1 Introduction

Gastrointestinal foreign body ingestion and obstruction is a commonly encountered condition in small animal veterinary medicine. This affliction can be treated medically, endoscopically, or surgically and endoscopic and/or surgical intervention is frequently required for resolution (1, 2). Postoperative management can be complicated by local and systemic inflammation, supraphysiologic intraluminal pressure, and intestinal wall ischemia which can result in intestinal dilation, ileus, and in some cases, loss of tissue viability and perforation (3). Postoperative management can also be challenging with potential complications after surgery including but not limited to upper gastrointestinal signs including vomiting and regurgitation which can predispose to esophagitis, aspiration pneumonia, and other sequelae; body wall hernia; surgical site infection; and most importantly intestinal dehiscence resulting in septic peritonitis, with reported rates of 0–12% (3).

Early enteral nutrition contributes to improved outcomes in the treatment of numerous diseases in small animal veterinary medicine (4–7). In human medicine this benefit has been documented, and well-established guidelines for nutritional management such as the ASPEN and ESPEN directives have existed for more than 20 years. Despite this, there is a large deficit in nutritional management protocols in the treatment of critically ill veterinary patients, and while there are some sporadic studies demonstrating the benefits of enteral nutrition for certain illnesses (4–7), there are no documented nutritional directives or guidelines available for veterinary medicine. Moreover, it is still fairly common practice for small animal veterinary patients to be fasted for prolonged periods with certain illnesses and procedures (i.e., fasting for 12 to 24 h following gastrointestinal surgery or withholding food for certain periods of time after episodes of vomiting or dietary indiscretion).

Nasogastric feeding tubes (NGT) are often used to provide temporary nutritional support in the critically ill patient. They are the least invasive type of feeding tubes and carry the lowest risk, but do have several documented complications, most notable being pneumothorax, esophageal perforation, improper placement, and dislodgement (8, 9). An overlooked utility of the NGT is its diagnostic use. In human medicine, NGT are frequently utilized to determine gastric residual volume (GRV) for identification and quantification of delayed gastric emptying, ileus, and feeding intolerance (10, 11); although this practice is often applied in veterinary medicine, to the authors’ knowledge there are currently no veterinary publications investigating its potential benefits. In fact, one of most notable consequences of not monitoring GRV in human literature is an increased risk for vomiting (12). While high GRV in a critically ill patient could result in a delay of enteral nutrition in the ICU setting, there seems to be little correlation in humans between increased GRV and poor outcomes like aspiration, ventilator-associated pneumonia, and increased mortality rates (13–15).

Ileus is a common sequela from gastrointestinal foreign body obstruction. Diagnosing and quantifying ileus can be difficult, and a variety of methods have been utilized such as diagnostic imaging and measurement of GRV. In human studies, GRV of up to 200–500 mL can be tolerated without increased risk for vomiting, aspiration, or the need to delay enteral nutrition, depending on the underlying disease process (16–18). In fact, recent ASPEN guidelines recommend that enteral nutrition be continued for most critically ill patients unless GRV is >500 mL or there are other clinical signs of intolerance to enteral nutrition (19).

The primary objective of this retrospective study is to evaluate postoperative GRV in dogs and cats when NGT was placed for clinical management after surgical gastrointestinal foreign body removal. A secondary goal of this study was to determine if there was a correlation of NGT placement, GRV measurement, or lack thereof to any undesired clinical outcomes.

## 2 Materials and methods

### 2.1 Study design and data collection

A retrospective review of medical records at a single institution emergency and referral specialty hospital was performed. Patients were identified through search of electronic medical records and dogs and cats that underwent gastrointestinal surgery for foreign body removal between August 2020 and November 2023 were included. Diagnosis of gastrointestinal foreign body obstruction was made based on diagnostic imaging (abdominal radiographs and/or abdominal ultrasound) and confirmed intraoperatively. Exclusion criteria consisted of missing or incomplete medical records, patients discharged within the first 8 h after surgery, and patients with other significant medical conditions potentially complicating their recovery such as incidental visceral tumors encountered during exploratory laparotomy, diaphragmatic hernia, gastric dilatation and volvulus, and owner-requested medical boarding prolonging the total time in hospital.

The medical record of each eligible case was reviewed for data collection. Recorded data included signalment, body weight, and body condition score. Pre-admission clinical signs and duration of vomiting, regurgitation, anorexia, and diarrhea were recorded. The surgery time and date were recorded as well as intraoperative findings, location of foreign body, and surgical procedures performed. It was also documented if a NGT was placed for case management as well as the timing of placement relative to surgery. All medications administered during hospitalization were documented, particularly any medications that influence gastrointestinal motility such as prokinetics and opioids. Any gastrointestinal signs (i.e., vomiting, regurgitation, diarrhea) occurring in the postoperative period were also recorded as well as any postoperative complications, and length of hospital stay.

The time to start of nutrition was also documented; whether a pet was eating on their own or if enteral nutrition was utilized was recorded including the type of diet administered for enteral nutrition. There was no standardized protocol for supplemental enteral nutrition; this was entirely up to clinician preference and there were various protocols utilized ranging from bolus feeding every 6 h to trickle feeding as a constant rate infusion. A variety of different diets were used including commercial liquid diets and canned food blended with water. Initial feeding rates ranged from 25 to 33% of the resting energy requirement and sometimes were increased incrementally depending on patient tolerance, time in hospital, and voluntary food intake. The pet was considered to be eating on their own after voluntarily consuming any amount, though the voluntary caloric intake could not be ascertained from the medical records. Measurement of GRV over time was documented in 6-h intervals starting from the time of surgery or the time of NGT placement if placed in the postoperative period. If a patient had an NGT placed, aspiration and GRV measurement was performed from the time of placement every 4–6 h depending on clinician preference. For all patients, the GRV fluid aspirated was discarded after removal and quantification.

### 2.2 Statistical analysis

Summary statistics were calculated for categorical variables as percentages; and for numerical variables as either mean ± SD or median (range) depending on parametric or nonparametric distributions, respectively, as assessed by the Shapiro–Wilk test. GRV was quantified by summing the retrieved volumes over the first 6-, 12-, or 24-h post-surgery, and then normalized for body mass by dividing the GRV sum by the patient’s admission weight in kg. Associations between categorical variables were assessed by the chi-square test of independence unless 20% or more of expected frequencies were <5. In those situations, associations were assessed by Fisher’s exact test. Comparisons between groups of independent numerical data were made by the Wilcoxon rank sum test due to the nonparametric numerical distribution in one or both groups. Data distributions for numerical data were graphed as box-and-whisker plots or as violin plots.

Pharmacological interventions were assessed statistically by creating binary variables for usage (yes/no) for each drug. A binary normalized variable of GRV per period of time divided by weight was created after determining an optimal discriminatory cut-off value in bivariate analysis. Associations between independent variables and the occurrence of either vomiting/regurgitation or diarrhea as outcomes of interest were assessed by backward stepwise multivariable logistic regression, removing variables from the model if *p* > 0.15. Odds ratios and 95% confidence intervals (CIs) were calculated for independent variables by exponentiation of their coefficients from logistic regression. A *p*-value of <0.05 was considered statistically significant.

## 3 Results

Within the study period, 525 patients were surgically treated for gastrointestinal foreign body obstruction. There was a total of 56 exclusions: 30 due to complicating co-morbidities occurring in the perioperative period, 15 for incomplete medical records, 10 for early discharge (<8 h after surgery), and 1 for prolonged hospital stay because of medical boarding.

Of the 469 patients included in the study, 376 (80.2%) were dogs and 93 (19.8%) were cats. There were 210 patients (44.8%) with NGT placed [177 (84.3%) dogs and 33 (15.7%) cats] and 259 (55.2%) had no NGT placed [199 (76.8%) dogs and 60 (23.2%) cats]. The median age at presentation was 2.25 year (range 0.12–15.8 year). Patients with NGT were slightly older than those without [3.0 (0.2–13.7) vs. 2.0 (0.12–15.8) respectively; *p* = 0.010]. The median body weight at presentation was 18.6 kg (range 0.43–74.0 kg), and patients with NGT were heavier than those without [20.4 (2.2–74.0) vs. 16.0 (0.43–70.1) respectively; *p* = 0.015]. In the study population, males were more prevalent, with 252 castrated males (53.7%), 53 intact males (11.3%), 125 spayed females (26.6%), and 24 intact females (5.1%). There were 81 breeds, with mixed breed dogs most common (32.2%). Sex, neuter status, and breed did not significantly differ between NGT groups.

In the study population there were 245 gastrotomies, 300 enterotomies, and 27 enterectomies. Some patients had multiple procedures performed. Likelihood of NGT placement did not differ in patients having gastrotomy or enterotomy (*p* = 0.956 and 0.478, respectively) but was significantly greater in patients with enterectomies [23/27 (85.2%); *p* < 0.001].

Postoperative gastrointestinal signs were noted regardless of NGT placement with no statistical significance in crude rates between the two groups (*p* = 0.118). 68 patients (14.5%) experienced vomiting or regurgitation (V/R) in the postoperative period [14.3% (30/210) in the NGT group vs. 14.7% (38/259) in the group without NGT]. 58 patients (12.4%) experienced diarrhea after surgery [15.7% (33/210) in the NGT group vs. 9.6% (25/259) in the non-NGT group].

Medications administered in the perioperative period include intravenous fluids (plasmalyte-R, lactated ringers solution, 0.9% sodium chloride, and fluid additives such as potassium chloride and dextrose), opioid pain medications (fentanyl, methadone, hydromorphone, and butorphanol), oral pain medications (gabapentin, tramadol), locoregional anesthetics in the form of transversus abdominus plane blocks and liposomal bupivacaine, antibiotics (ampicillin sulbactam, amoxicillin/clavulanic acid, enrofloxacin, clindamycin), gastrointestinal support drugs (maropitant, pantoprazole, ondansetron), prokinetics (erythromycin and metoclopramide), appetite stimulants (capromorellin and mirtazipine), and other medications such as sedatives (acepromazine, dexmedetomidine, and trazodone), glucocorticoids (prednisone and prednisolone), and furosemide. Metoclopramide administration was markedly associated with V/R in the whole study population (OR 11.47; 95% CI: 5.70–23.10; *p <* 0.001) while patients given methadone exhibited significantly less V/R (OR 0.48; 95% CI: 0.26–0.91; *p =* 0.024) than those administered other opioids. When statistical analysis controlled for drug administration, odds of V/R were reduced approximately 50–60% for patients with NGT (OR 0.43; 95% CI: 0.21–0.87; *p* = 0.018) compared to those without NGT.

**Figure 1:**
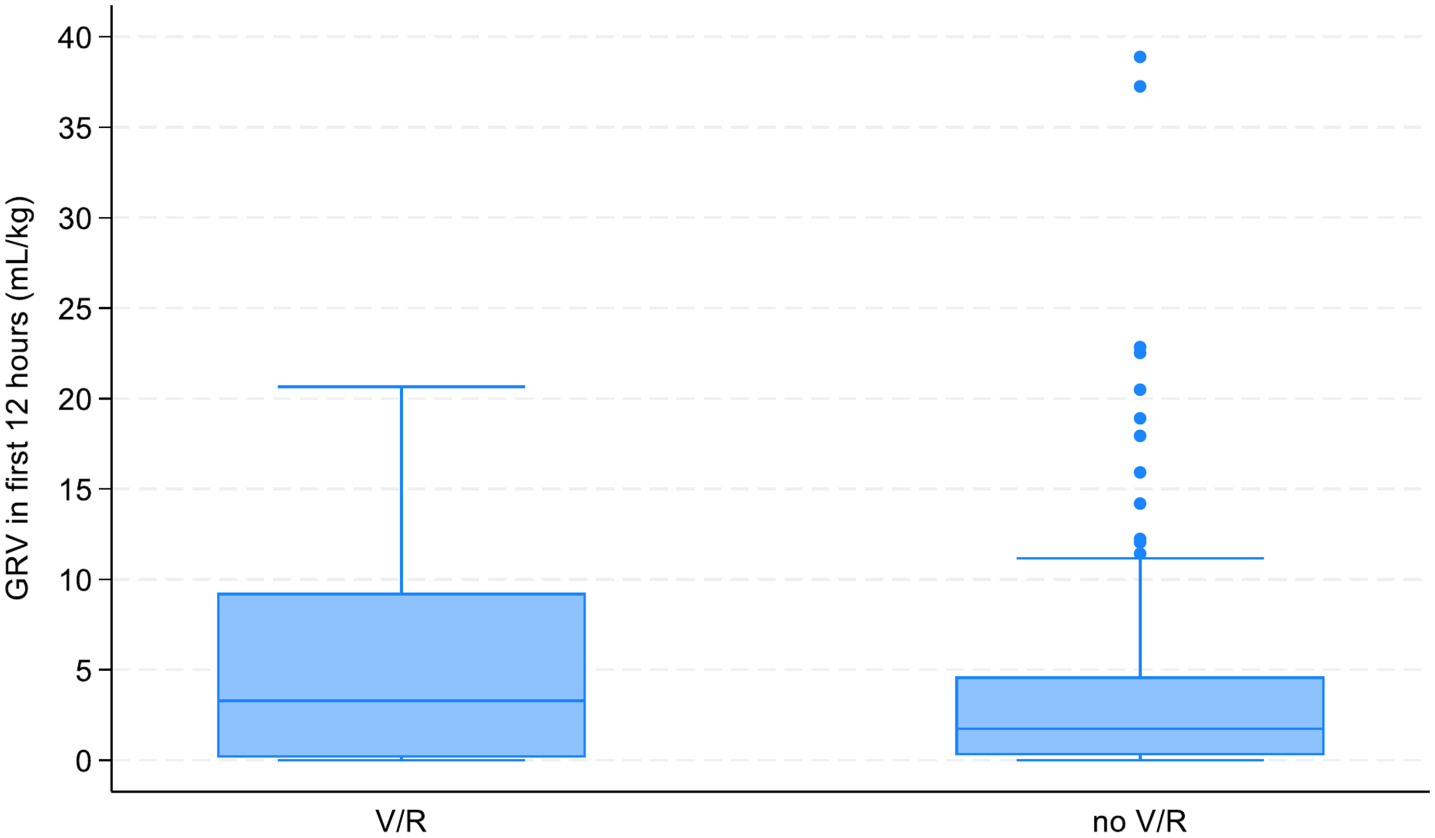
Gastric residual volumes (GRV) removed from nasogastric tubes of canine and feline patients in the first 12 h postoperative gastrointestinal foreign body removal surgery. The *x*-axis establishes patients in two groups, those who had one or more occurrence(s) of vomiting or regurgitation (V/R) (*n* = 19) and those who did not experience any vomiting or regurgitation (no V/R) (*n* = 164). The *y*-axis indicates the GRV in mL/kg. Data is shown as median line within box (IQR) and whiskers (range) unless values were >1.5× the IQR.

**Figure 2:**
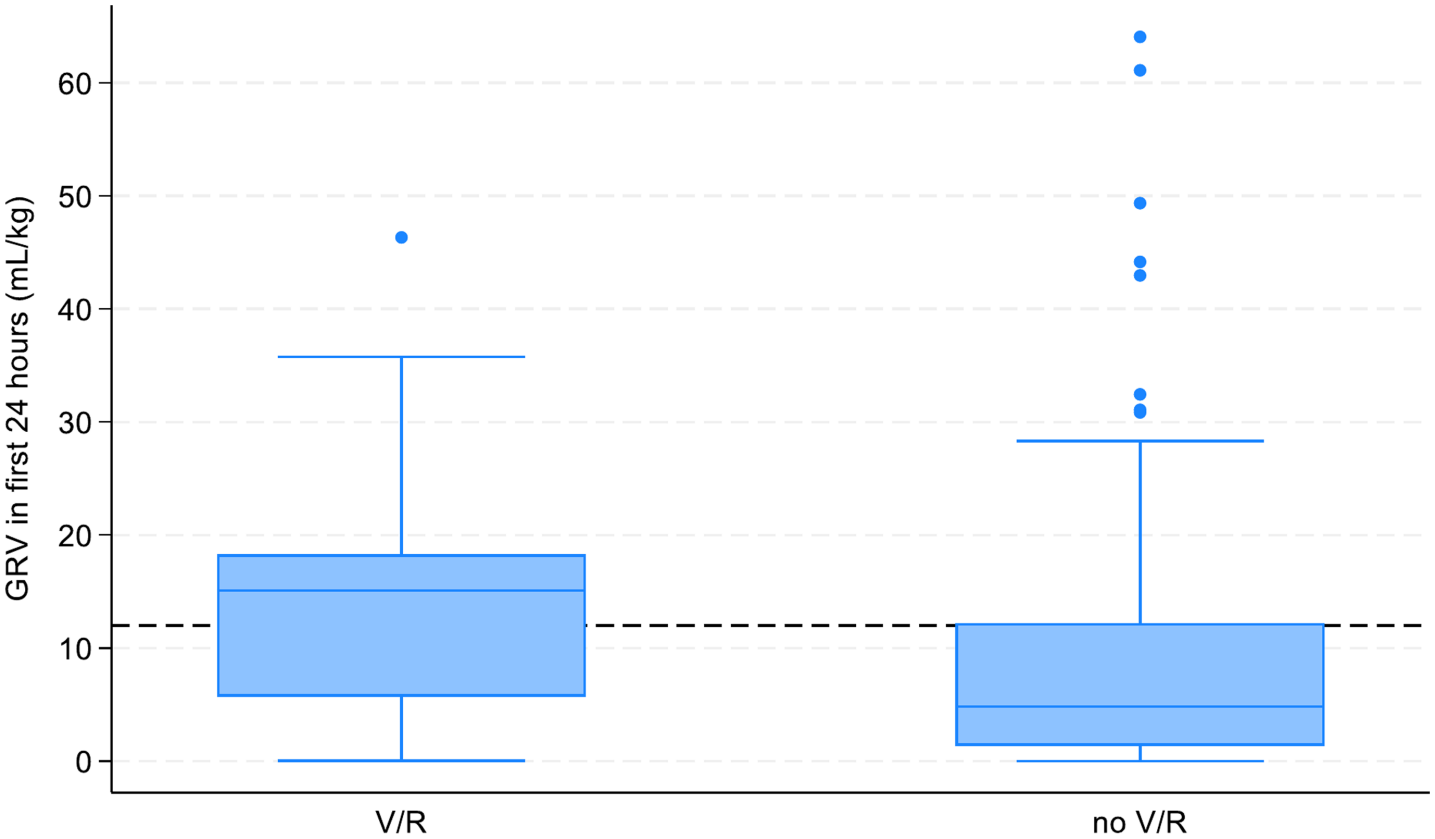
Gastric residual volumes (GRV) removed from nasogastric tubes of canine and feline patients in the first 24 h following gastrointestinal foreign body removal surgery in canine and feline patients, separated by the occurrence (*n* = 18) or absence (*n* = 113) of vomiting/regurgitation (V/R) in the postoperative period. Cut-off value of 12 mL/kg shown as horizontal dashed line. See Figure 1 for key.

Of the 210 patients within the NGT group, 183 had complete data for GRV calculation for the first 12 h and 131 had data for the first 24 h after surgery. Total GRV for the first 12 h after surgery [median (IQR)] was 2.0 mL/kg (0.3–5.1 mL/kg) with a range of 0.0–38.9 mL/kg (Figure 1). Total GRV reported in the first 24 h after surgery was 5.4 mL/kg (IQR: 1.4–15.1 mL/kg) with a range of 0.0–64.1 mL/kg (Figure 2). Only 24 patients (21 dogs and 3 cats) had GRV data for >24–48 h post-surgery. Total GRV for the second 24 h after surgery for these patients was 7.0 mL/kg (IQR: 0.6–25.0 mL/kg) with a range of 0.0–79.5 mL/kg.

In the first 12 h after surgery, V/R was reported in 10.4% (19/183) of NGT patients but there was no GRV cut-off volume which significantly distinguished between patients with or without V/R. When using the most significant GRV cut-off volume of 9 mL/kg within in the first 12-h period postoperative, 19.2% of NGT group patients with GRV of >9 mL/kg (5/26) experienced V/R while only 8.9% of patients with GRV ≤ 9 mL/kg (14/157) experienced V/R (*p* = 0.110) (Figure 1).

In the first 24 h after surgery, GRV > 12 mL/kg was most significantly associated with V/R postoperatively. V/R was reported in 27.5% (11/40) of NGT patients with GRV > 12 mL/kg, but in only 7.7% (7/91) of patients with GRV ≤ 12 mL/kg (*p* = 0.002) (Figure 2). In multivariable analysis, however, the odds of V/R associated with GRV > 12 mL/kg was confounded by postoperative drug administration. Inclusion of metoclopramide and methadone into the analysis for V/R reduced the odds ratio of GRV > 12 mL/kg from 4.55 to 3.25 (*p* = 0.058).

Overall, cats were less likely than dogs to have feeding tubes placed postoperatively [35.5% (33/93) vs. 47.1% (177/376), respectively; *p* = 0.044]. This significant difference may be attributed to clinician preference, anatomical differences making placement more difficult, or the timing of presentation within the study which is further elaborated upon in the discussion. Regardless of NGT placement and feeding method, cats were significantly less likely to experience V/R compared to dogs [1.1% (1/93) vs. 17.6% (66/376), respectively; *p* < 0.001]. There were two cats as outliers within the NGT group that did not vomit or regurgitate despite excessively large total volume GRV (173 and 155 mL respectively) in the first 24 h after surgery. Diarrhea was also less frequent in feline patients [5.4% (5/93)] than in canine patients [14.1% (53/376)] (*p* = 0.022).

The overall reported complication rate associated with feeding tube placement within the NGT group was 6/210 = 2.9% (95% CI, 1.1–6.1%). Documented complications included dislodgement (*n* = 3), iatrogenic pneumothorax (*n* = 2), and difficulty with placement (*n* = 1). For the majority of patients NGT placement was performed while under anesthesia for surgery either just before, during, or immediately after the procedure (88.1%), however, some patients had NGT placed prior to surgery or in the postoperative period due to intractable V/R. Patients with NGT had significantly longer length of hospitalization until discharge postoperatively (median 32.5 h, range: 10–136 h) compared to patients without NGT (median 22 h, range: 9–73 h; *p* < 0.001).

## 4 Discussion

Nasogastric feeding tubes are an important aspect of nutritional management in the postoperative and critical care setting. Human medicine has established guidelines for nutritional management and NGT usage (11–15) including maximum tolerated GRV for certain conditions such as critically ill burn victims and neurologically compromised patients (16–18). Though some information can be carried over from the human guidelines, there is still an absence of knowledge in veterinary medicine relating to utilization of GRV as well as enteral nutrition protocols in veterinary medicine. The present study aims to improve upon that knowledge gap by providing information regarding usage of the NGT and GRV in the postoperative setting after gastrointestinal foreign body surgery.

The primary goal with this project is to provide clinicians utilizing GRV with some criterion to help guide postoperative treatment protocols after foreign body surgery. The biggest difference between GRV evaluation in human and veterinary medicine is the wide variability in patient size and body weight in animals which affects potential gastric reservoir volumes between patients. For this reason, the authors have chosen to use volume compared to body weight rather than a single total volume measurement as is seen in the majority of human literature, however, it is important to note that in human literature, both quantification methods have been utilized (17, 18, 20).

Prior to acquisition of these results, the primary author had utilized a GRV of 5 mL per kilogram of body weight as a cut-off for considering when to start additional gastrointestinal support interventions in the postoperative period. The data in this study demonstrates that a GRV of >12 mL/kg within the first 24-h period after gastrointestinal foreign body surgery is associated with increased risk for vomiting and regurgitation (Figure 2). Using the current data to extrapolate, GRV of greater than 3 mL/kg within a 6-h period could increase the risk for vomiting and regurgitation and should be considered as a cut-off or call parameter value for when to consider adding prokinetics or other gastrointestinal supportive care, especially if this is a consistent trend over time.

One major factor that could play a role in the development of ileus and regurgitation after gastrointestinal foreign body surgery is location of the foreign object or obstruction and the type of surgery performed. Except for enterectomies, there was no statistical difference between surgical procedures between the two groups within this study. As a retrospective study, it is probable that there was selection bias making NGT placement more likely for those patients having enterectomies over other gastrointestinal procedures, as clinicians may be more likely to use an NGT after a surgery that is more prone to postoperative complications. Other factors that may contribute to delayed gastric emptying and ileus in the postoperative period include physiologic changes that occur secondary to intestinal obstruction, anorexia and decreased food intake, as well as anesthetic and medication-related factors.

One relevant, but unexpected finding of this project was the lower rate of regurgitation seen across all groups in animals that were administered methadone as an opioid pain medication. This is supportive of previous literature (21) and clinical experience that methadone administration carries less risk for nausea, vomiting, and regurgitation. Based upon this observation, the authors recommend the use of methadone, if possible, over other opioid for perioperative pain management with gastrointestinal foreign body removal, though additional prospective clinical study is necessary to better understand this finding. The correlation between metoclopramide and increased incidence of V/R was likely due to the fact that this medication was typically the first line choice at this hospital to treat these clinical signs after gastrointestinal foreign body surgery because of its prokinetic effects.

Another unexpected finding was the significantly low rate of postoperative vomiting and regurgitation exhibited by the cats in this study. This may simply be related to the fact that cats composed a smaller population, however, this finding was statistically significant (*p* = 0.044). This finding supports the authors’ clinical experience that cats with foreign body obstructions are less prone to regurgitation in general compared to dogs. Additional study is needed to better elucidate this finding in cats undergoing gastrointestinal surgery for foreign body removal.

Presently, there are no accepted guidelines on how frequently a NGT should be aspirated and what to do with the gastric fluid after removal. There is some debate on whether to re-introduce this gastric residual fluid after quantification or to discard it. Current literature shows no significant risks with discarding the GRV after aspiration (22, 23). In this study, the GRV fluid was discarded for all patients. The authors believe that discarding rather than returning the gastric fluid may help to control clinical signs of delayed gastric emptying and enteral nutrition intolerance, although additional prospective evaluation is needed to better understand the potential benefits and risks.

Additionally, it is worth noting that in human medicine, small-bore NGT [8 Fr] have been found to be more easily clogged, which can reduce the accuracy of the GRV measurement (24). A significant portion of NGT used in veterinary medicine are 8 Fr or smaller. This could have affected or lowered the gastric residual volumes achieved within the current study population making it a potential limitation within this study.

Limitations of this study include the retrospective nature of the study design. This inherently introduces inconsistencies with non-standardized medication protocols and variations in rates of NGT placement or GRV sampling frequency due to differences in clinician preference. Additionally, quantity of voluntary food intake was not consistently well-documented in the records, making it impossible to determine significance of voluntary nutritional intake and how it may have affected GRV or postoperative vomiting and regurgitation. The decision to pursue this project was made when a change in the clinical practice recommendation was seen at this institution to begin incorporating prophylactic NGT placement for postoperative management in patients undergoing foreign body surgery, both for management of postoperative ileus as well as to provide early enteral nutritional support.

Another major limitation of this study is that GRV quantification can be subject to sampling error, human error, and other inconsistencies. The biggest limitation in terms of GRV quantification in this study is the non-standardized timing of NGT aspiration, however, all patients included in this study had their GRV measured either every 4 or every 6 h consistently; timing was based solely on clinician preference. Other limitations include non-standardized anesthesia and postoperative drug or treatment protocols within the institution as certain drug choices may have affected ileus, GRV, appetite, and may have caused or prevented V/R. Future studies should be prospective and directed at determining normal GRV in healthy populations of dogs and cats as well as use of standardized anesthetic, postoperative treatment, and nutritional protocols to decrease the impact of these variables on results.

While there is still much to be accomplished in developing nutritional protocols for postoperative and critical care sectors of veterinary medicine, our hope is that these observations provide helpful information for managing postoperative gastrointestinal surgical patients with NGT for both diagnostic and treatment purposes. Nasogastric tubes are relatively inexpensive, easy to place—especially while under general anesthesia, and generally well-tolerated in small animals. Future studies will be directed at determining the overall impact of enteral nutrition (including timing of administration and type of nutrition administered) on GRV, postoperative ileus, and patient outcomes after gastrointestinal foreign body surgery.

## 5 Conclusion

In conclusion, nasogastric feeding tubes are low-risk, minimally invasive feeding tubes with both diagnostic and therapeutic benefits and should be considered standard of care for any veterinary patient undergoing major gastrointestinal surgery. Quantification and monitoring of trends in gastric residual volume in postoperative and critically ill patients can be a helpful diagnostic tool in predicting vomiting and regurgitation. Further studies are needed to determine normal ranges for gastric residual volume, as well as the impact of intestinal obstruction, enteral nutrition, and medications on delayed gastric emptying and ileus; however, application of a maximum tolerated gastric residual volume of >3 mL/kg in a 6-h period or >12 mL/kg in the first 24-h period after gastrointestinal foreign body surgery for dogs and cats could help to anticipate vomiting and regurgitation, particularly after foreign body removal surgery.

## Data Availability

The raw data supporting the conclusions of this article will be made available by the authors, without undue reservation.

## References

[1] Carrillo AJ McCord MA Dickerson VM. Clinical features and outcomes of dogs with attempted medical management for discrete gastrointestinal foreign material: 68 cases (2018–2023). J Am Vet Med Assoc. (2024) 262:1251–8. doi: 10.2460/javma.24.01.0050, 38823414

[2] Prettegiani B Maritato K. Comparison of removal of intestinal foreign bodies using orogastric retrieval techniques versus gastrotomies in dogs and cats. J Small Anim Pract. (2025) 66:243–7. doi: 10.1111/jsap.13827, 39799984

[3] Mullen KM Regier PJ Ellison GW Londoño L. The pathophysiology of small intestinal foreign body obstruction and intraoperative assessment of tissue viability in dogs: a review. Top Companion Anim Med. (2020) 40:100438. doi: 10.1016/j.tcam.2020.100438 32690289

[4] Liu DT Brown DC Silverstein DC. Early nutritional support is associated with decreased length of hospitalization in dogs with septic peritonitis: a retrospective study of 45 cases (2000–2009). J Vet Emerg Crit Care. (2012) 22:453–9. doi: 10.1111/j.1476-4431.2012.00771.x, 22928749

[5] Will K Nolte I Zentek J. Early enteral nutrition in young dogs suffering from Haemorrhagic gastroenteritis. J Vet Med. (2005) 52:371–6. doi: 10.1111/j.1439-0442.2005.00745.x, 16109106

[6] Mohr AJ Leisewitz AL Jacobson LS Steiner JM Ruaux CG Williams DA. Effect of early enteral nutrition on intestinal permeability, intestinal protein loss, and outcome in dogs with severe parvoviral enteritis. J Vet Intern Med. (2003) 17:791–8. doi: 10.1111/j.1939-1676.2003.tb02516.x, 14658714 PMC7166426

[7] Economu L Chang Y Preistnall SL Kathrani A. The effect of assisted enteral feeding on treatment outcome in dogs with inflammatory protein-losing enteropathy. J Vet Intern Med. (2021) 35:1297–305. doi: 10.1111/jvim.16125, 33931908 PMC8163126

[8] Yu MK Freeman LM Heinze CR Parker VJ Linder DE. Comparison of complication rates in dogs with nasoesophageal versus nasogastric feeding tubes. J Vet Emerg Crit Care. (2013) 23:300–4. doi: 10.1111/vec.12048, 23621520

[9] Odunayo A Hoen M Wolf J Marshall K Osterbur K Maxwell K. Outcomes, including death, in dogs with pneumothorax following nasogastric feeding tube misplacement in the tracheobronchial tree: 13 cases (2017–2022). J Am Vet Med Assoc. (2023) 261:1–7. doi: 10.2460/javma.22.12.0585, 37146975

[10] Ukleja A. Altered GI motility in critically ill patients: current understanding of pathophysiology, clinical impact, and diagnostic approach. Nutr Clin Pract. (2010) 25:16–25. doi: 10.1177/0884533609357568 20130154

[11] Tanguy M Seguin P Mallédant Y. Bench-to-bedside review: routine postoperative use of the nasogastric tube—utility or futility? Crit Care. (2007) 11:201–9. doi: 10.1186/cc5118, 17214909 PMC2151862

[12] Wang Z Ding W Fang Q Zhang L Liu X Tang Z. Effects of not monitoring gastric residual volume in intensive care patients: a meta-analysis. Int J Nurs Stud. (2019) 91:86–93. doi: 10.1016/j.ijnurstu.2018.11.005, 30677592

[13] McClave SA Lukan JK Stefater JA Lowen CC Looney SW Matheson PJ . Poor validity of residual volumes as a marker for risk of aspiration in critically ill patients. Crit Care Med. (2005) 33:324–30. doi: 10.1097/01.ccm.0000153413.46627.3a, 15699835

[14] Diamond SJ Medici V Rice TW Miller K. Should we stop using gastric residual volumes? Curr Nutr Rep. (2015) 4:236–41. doi: 10.1007/s13668-015-0129-3

[15] Barkhordari M Jahani S Soltani F Molavynejad S Maraghi E. Effect of tubular feeding with the measurement of gastric residual volume on ventilator associated pneumonia. Tanaffos. (2021) 20:319–26. 36267927 PMC9577209

[16] Pham CH Collier ZJ Garner WL Kuza CM Gillenwater TJ. Measuring gastric residual volumes in critically ill burn patients—a systematic review. Burns. (2019) 45:509–25. doi: 10.1016/j.burns.2018.05.011, 29914737

[17] Pinilla JC Samphire J Arnold C Liu L Thiessen B. Comparison of gastrointestinal tolerance to two enteral feeding protocols in critically ill patients: a prospective, randomized controlled trial. JPEN. (2001) 25:81–6. doi: 10.1177/014860710102500281, 11284474

[18] Liu F Liu G Sun R Wang J Li M Gong L . Comparison of two different threshold values for the measurement of gastric residual volume on enteral nutrition support in the neurocritically ill patients. Front Nutr. (2022) 9:1–7. doi: 10.3389/fnut.2022.871715, 35799592 PMC9253574

[19] McClave SA Tayler BE Martindale RG Warren MM Johnson DR Braunschweig C . Guidelines for the provision and assessment of nutrition support therapy in the adult critically ill patient. JPEN. (2016) 40:159–211. doi: 10.1177/0148607115621863, 26773077

[20] Flynn D. N. Doyal A. Schoenherr J. W. Gastric ultrasound. In: StatPearls. Treasure Island: StatPearls Publishing; (2025). Available online at: https://www.ncbi.nlm.nih.gov/books/NBK580524/# (accessed February 20, 2023)

[21] Kerr CL Swanton WE. Anesthesia update—incorporating methadone into companion animal anesthesia and analgesic protocols: a narrative review. Can Vet J. (2023) 64:1058–65. doi: 10.4148/2560-7990.2727, 37915778 PMC10581351

[22] Wen Z Xie A Peng M Bian L Wei L Li M. Is discard better than return gastric residual aspirates: a systematic review and meta-analysis. BMC Gastroenterol. (2019) 19:113–9. doi: 10.1186/s12876-019-1028-7, 31253100 PMC6599274

[23] Chih A Rudloff E Waldner C Linklater AKJ. Incidence of hypochloremic metabolic alkalosis in dogs and cats with and without nasogastric tubes over a period of up to36 hours in the intensive care unit. J Vet Emerg Crit Care. (2018) 28:244–51. doi: 10.1111/vec.12720 29727526

[24] Powell KS Marcuard SP Farrior ES Gallagher ML. Aspirating gastric residuals causes occlusion of small-bore feeding tubes. JPEN. (1993) 17:243–6. doi: 10.1177/0148607193017003243, 8505829

